# Utility of Prognostic Nutritional Index and Systemic Immune‐Inflammation Index Inpatients With Sudden Sensorineural Hearing Loss: A Large Prospective Cohort Study

**DOI:** 10.1002/iid3.70217

**Published:** 2025-06-17

**Authors:** Xu Zhang, Junyi Wu, Wentao Zhang, Maohua Wang, Bing Guan, Qiao Jiang, Chunping Yang

**Affiliations:** ^1^ Department of Otorhinolaryngology Head and Neck Surgery, The Second Affiliated Hospital, Jiangxi Medical College Nanchang University Nanchang Jiangxi China; ^2^ Department of Otolaryngology‐Head and Neck Surgery Northern Jiangsu People's Hospital Affiliated to Yangzhou University Yangzhou Jiangsu China; ^3^ Department of Otolaryngology, Head and Neck Surgery The First Affiliated Hospital of Anhui Medical University Hefei Anhui China; ^4^ Department of Otolaryngology, Head and Neck Surgery The First People's Hospital of Foshan, Hearing and Balance Medical Engineering Technology Center of Guangdong Foshan Guangdong China; ^5^ Department of Neurology Deyang Fifth Hospital Deyang Sichuan China

**Keywords:** immune nutritional level, prognostic nutritional index, sudden sensorineural hearing loss, systemic immune inflammatory index

## Abstract

**Background:**

This study mainly explores the correlation between prognostic nutritional index (PNI) and systemic immune inflammatory index (SII) and sudden sensorineural hearing loss (SSNHL).

**Methods:**

In this study, 150 SSNHL patients (average age 48.98 ± 16.45 years; 48.92% male, 50.93% female) were categorized into effective and ineffective treatment groups. Additionally, 150 healthy volunteers (average age 49.49 ± 9.75 years; 51.08% male, 49.07% female) served as the control group. Baseline characteristics, clinical data, and laboratory results were collected, and SII and PNI were calculated for analysis. Linear correlation, logistic regression, and receiver characteristic curve analyses were conducted to explore the link between immune nutrition levels and SSNHL.

**Results:**

In the SSNHL group, PNI was significantly lower, while SII, platelet, and neutrophil counts were notably higher compared to controls (*p* < 0.001). Logistic regression identified low PNI (OR = 0.878), high SII (OR = 1.005), and elevated neutrophils (OR = 1.758) as predictors of SSNHL. Data comparison showed higher nutritional levels in the effective treatment group than in the ineffective group. Logistic regression indicated that low PNI (OR = 1.075) and high SII (OR = 1.004) were strongly linked to treatment outcomes.

**Conclusion:**

There is a relationship between the body's immune nutrition level and the occurrence and development of SSNHL. Lower PNI and higher SII are associated with the occurrence of SSNHL and poor outcomes. However, further research into the underlying mechanisms is needed.

## Graphical abstract

In this study, we investigated the relationship with human inflammatory status and nutritional level with the occurrence and prognosis of SSNHL patients. Lower PNI values and higher SII values may promote the occurrence of SSNHL, while higher PNI values and lower SII values may suggest a better prognosis for SSNHL. Our findings will benefit clinicians in the disease management of SSNHL patients and in the development of individualized treatment plans.

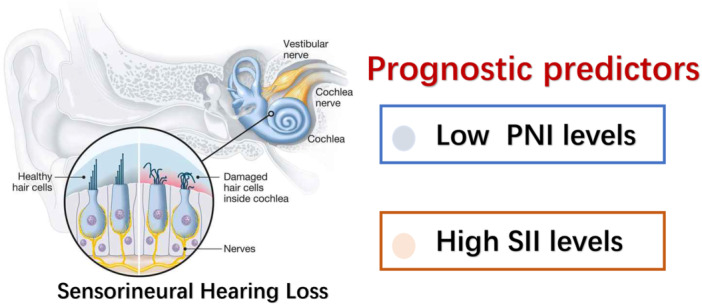

## 1 Introduction

Sudden sensorineural hearing loss (SSNHL) represents a prevalent emergency condition within the field of otolaryngology, characterized by an abrupt and unexplained decline in auditory function occurring within a 72‐h timeframe. This condition is specifically defined by a hearing loss of at least 30 decibels across three contiguous frequencies as measured on an audiogram [1].

Studies indicate that the annual incidence of SSNHL is rising globally. In China, it ranges from 0.005% to 0.02%, while in the U.S., it is 27 cases per 100,000 people, increasing with age. Japan reported 27.5 cases per 100,000 in 2001, with an upward trend, and Germany saw a rise from 20 cases per 100,000 in 2004 to 160–400 cases by 2011. This growing prevalence and associated disabilities challenge healthcare systems significantly [2, 3, 4, 5].

The cause of sudden deafness is still unclear. It is generally believed that the cause is related to viral infection, vascular disease, autoimmune disease and others [6]. In addition, cochlear endolymphatic hydrops is also a major cause of SSNHL, especially in younger patients [7]. Therefore, exploring the clinical characteristics [8] and related risk factors [9] of sudden deafness is of great significance for clinical physicians to analyze the pathogenesis of SSNHL and formulate prevention and treatment measures.

In the past few years, the concept of pathological activation of cellular stress pathways has attracted more attention, especially concerning the vital role of immune inflammation in the development of sudden sensorineural hearing loss [10]. The inner ear mainly receives blood from a single terminal artery, and the cochlear hair cells have high oxygen requirements but low tolerance for low‐oxygen conditions. Inflammation of the immune system can impact the microcirculation in the inner ear, leading to hearing loss [11]. In all SSNHL subtypes, inner ear microcirculatory disorders are an important etiologic factor [4, 12]. Inflammatory response is often positively correlated with disease severity and poor prognosis [13]. The most common indicators of inflammatory status include albumin, neutrophils, and neutrophil‐to‐lymphocyte ratio (NLR). In addition, the body's immune and nutritional status play an important role in hearing loss and recovery [14]. Based on serum albumin and lymphocyte counts, the prognostic nutritional index (PNI) reflects the patient's immune and nutritional status [15]. Malnutrition and immunosuppression are often indicated by low PNI values, which can also serve as predictors for the severity and prognosis in patients with inflammatory diseases [16, 17]. Additionally, the systemic immune inflammatory index (SII) is another important parameter that assesses the immune and inflammatory status of the body, calculated from neutrophil, platelet, and lymphocyte counts [18]. Elevated SII values are often indicative of a more intense inflammatory response and are valuable in forecasting the severity and prognosis of tumors, inflammatory diseases, and other conditions [19, 20]. Recent study has demonstrated that higher SII values often correspond with a less favorable prognosis for SSNHL [21].

Despite the limited number of studies examining the influence of the SII and PNI on the severity of hearing loss and prognostic outcomes in patients with SSNHL, their clinical significance and potential utility warrant further investigation. This prospective study aims to elucidate the clinical relevance of immune‐inflammatory markers, specifically the SII and PNI, in individuals with SSNHL. The research endeavors to evaluate metabolic levels, immune function, and tissue repair mechanisms, thereby providing a theoretical basis for the prevention and management of SSNHL. Furthermore, we hypothesize that the immune‐nutritional status, quantified by SII and PNI, demonstrates an independent association with both the risk of SSNHL occurrence and treatment outcomes, and may serve as effective biomarkers for clinical prognosis. By integrating these indices into routine assessments, our findings aim to enhance early risk stratification and personalized therapeutic strategies for SSNHL patients.

## 2 Methods

### 2.1 Study Subjects

We conducted a prospective study of patients diagnosed with SSNHL in the School of Clinical Medicine of Yangzhou University from February 2023 to April 2024. The sample inclusion criteria were as follows: ① Age 18 and above; ② Sensorineural, sudden hearing loss within 72 h, at least 30 decibels across three consecutive frequencies on an audiogram ③ Unilateral onset; ④ No relevant treatments were administered before the onset of the condition. The exclusion criteria included: ① Patients with any concurrent diseases or injuries that could lead to hearing loss, such as Meniere's disease, chronic suppurative otitis media, central nervous system disorders, head trauma, or those who have used ototoxic medications; ② Patients with suspected space‐occupying lesions in the posterior cochlear region, as confirmed by cranial or middle ear MRI scans, or CT scans of the temporal bone. ③Patients with lesions of outer ear, middle ear, and central ear were confirmed by electrootoscope, tympanometry and Otoacoustic Emissions. ④ Previous HL and/or tinnitus; ⑤ Previous history of ear pathology and/or otologic surgery.

Finally, a total of 150 patients were enrolled in the study, with an equal number of 150 healthy individuals matched by sex and age included as the control group. These control subjects had normal hearing and were free of any diseases based on routine health check‐ups.

### 2.2 Data Collection

Comprehensive patient histories were gathered, covering baseline details like age, sex, BMI, and blood pressure at admission. Clinical data included the affected ear, symptoms (tinnitus, dizziness, ear fullness), and treatment duration. Before treatment, fasting blood samples were taken at 7 a.m. for routine hematological and lab tests. The absolute counts of peripheral blood. The following parameters were measured in peripheral blood: Glycosylated Hemoglobin (HbA1C), Creatinine (Cr), Albumin (Alb), Alanine Aminotransferase (ALT), Total Bilirubin (TBIL), Triglycerides (TG), High‐Density Lipoprotein (HDL), Low‐Density Lipoprotein (LDL), Total Cholesterol (TC), White Blood Cell count (WBC), Neutrophils (Gran), Aspartate Aminotransferase (AST), Lymphocytes (Lym), Mean Platelet Volume (MPV), Platelets (Plt), and Fibrinogen (FIB). Indirect Bilirubin (IBIL). Additionally, the SII and PNI values for each subject were calculated. The SII was derived using the formula: Neutrophils (N) × Platelets (P) ÷ Lymphocytes (L). The PNI was determined with the following formula: Serum albumin (g/L) + 5 × Total lymphocyte count (10^9/L)

We employed a forced‐entry method to retain all predefined variables, aligning with the study's exploratory aim. This approach ensures transparency and avoids data‐driven variable selection biases. Variables were selected based on: Clinical relevance (e.g., SII, PNI as primary predictors). Literature support (e.g., neutrophils and platelets linked to inflammation and microcirculation). Predefined hypotheses (e.g., immune‐nutritional markers as independent predictors).

### 2.3 Audiology Testing

Pure tone audiometry was performed on a cohort of 150 patients diagnosed with SSNHL both before and following treatment. The thresholds for bone and air conduction were measured at the frequencies of 250, 500, 1000, 2000, 4000, and 8000 Hz. The pure tone average (PTA‐4) was determined by calculating the average hearing threshold at frequencies of 250, 500, 1000, and 2000 Hz. Hearing is classified by the Chinese Medical Association using pure tone average (PTA) from 125 Hz to 8 kHz: normal hearing is less than 25 dB HL, mild hearing loss is 26–40 dB HL, moderate hearing loss is 41–60 dB HL, severe hearing loss is 61–80 dB HL, and very severe hearing loss exceeds 81 dB HL [9]. Treatment was considered effective if PTA improved by at least 15 dB or matched the unaffected ear. Patients were divided into “effective” and “ineffective” groups based on these outcomes.

### 2.4 Treatment Protocol

A total of 150 SSNHL patients underwent a 7–10 day comprehensive treatment regimen that included steroids and batroxobin. The detailed treatment plan was: 5 mg of dexamethasone was injected into the affected ear tympanic cavity once every other day, for a total of three times; intravenous infusion of methylprednisolone (1 mg/kg/day, for 3–5 days, and then the dose was reduced for the remaining days according to the improvement of hearing); intravenous infusion of nerve nutrition drugs (Ginaton) 70 mg/day; intravenous injection of batroxobin treatment (10 U of batroxobin was used for the first time, and then reduced to 5 U of batroxobin, once every other day, for a total of 1–3 times depending on the fibrinogen level).

### 2.5 Statistical Analysis Methods

Quantitative data are shown as median (Q1, Q3) for non‐normal distributions, and qualitative data as percentages. The Kolmogorov‐Smirnov test was used to assess the normality of the data. Data that were normally distributed with equal variances were evaluated using the *t*‐test, whereas the Mann‐Whitney U test was applied to non‐normal data. Since the PNI variable followed a normal distribution (*p* = 0.20), a *t*‐test was applied. For variables that did not follow a normal distribution (*p* < 0.05), the U test was utilized. Spearman's rank correlation explored relationships between PNI, SII, hearing loss, and recovery. A binary logistic regression model examined the link between PNI, SII, and SSNHL onset and prognosis, with the ROC curve assessing the model's predictive power. Variables such as age were dichotomized (< 50 vs. ≥ 50 years) based on clinical relevance and prior literature. Continuous variables (e.g., SII, PNI) were retained to maximize analytical precision. Variables (age, sex, BMI, SII, PNI, etc.) were predefined based on clinical hypotheses and literature support. A forced‐entry approach ensured comprehensive evaluation of immune‐nutritional markers while avoiding stepwise regression's risk of omitting key variables. Collinearity was mitigated via Variance Inflation Factor (VIF) testing (e.g., SII components were excluded to avoid redundancy). Statistical analysis and visualization were done with IBM SPSS 26.0, using a significance level of *α* = 0.05.

## 3 Results

### 3.1 Basic Situation of Participators

In this study, there were 150 patients diagnosed with SSNHL and 150 healthy control subjects. Table 1 outlines the main characteristics of the participants. The mean age of the SSNHL group was 51.00 years (interquartile range: 35.75, 61.00), whereas the control group had a mean age of 48.00 years (interquartile range: 42.00, 57.50). Analysis of the statistics indicated no significant age disparity between the two groups (*p* > 0.05). Furthermore, the groups did not show significant variations in gender distribution, body mass index (BMI), blood pressure, or laboratory values (*p* > 0.05). However, significant differences were identified in neutrophil and platelet counts (*p* < 0.01). The SSNHL group exhibited a significantly elevated systemic immune‐inflammation index (SII) compared to the control group (*p* < 0.001), whereas the control group demonstrated a marginally higher prognostic nutritional index (PNI) than the SSNHL group (*p* < 0.001). As detailed in Table 1, significant differences in immune‐nutritional markers (neutrophils, platelets, SII, and PNI) were observed between SSNHL patients and healthy controls (all *p* < 0.001). A comparative visualization of these parameters is provided in Supplementary Figure S1, further illustrating the distinct immune‐inflammatory and nutritional profiles of the SSNHL cohort.

**Table 1:** The comparison of the basic situation of participators in the study group and the control group.

	SSNHL group (*n* = 150)	Control group (*n* = 150)	*t*/*Z*/*χ* ^*2*^	*p* value
**Baseline characteristics**				
Age^a^	51.00 (35.75, 61.00)	48.00 (42.00, 57.50)	11049.50	0.789
BMI^a^	23.87 (21.98, 25.28)	23.15 (21.71, 25.22)	1.482	0.138
Gender^b^				
Male	68.00 (48.92%)	71.00 (51.08%)	0.12	0.728
Female	82.00 (50.93%)	79.00 (49.07%)		
DBP^a^	77.00 (71.00, 82.00)	78.00 (72.00, 80.00)	−0.434	0.664
SBP^a^	123.00 (112.00, 132.00)	122.00 (116.00, 128.00)	0.364	0.716
**Laboratory indicators**				
HDL^a^	1.32 (1.11, 1.56)	1.34 (1.05, 1.79)	−0.984	0.325
LDL^a^	3.12 (2.51, 3.44)	3.06 (2.64, 3.58)	−1.117	0.264
HbA1c^a^	5.70 (5.40, 6.33)	5.60 (5.40, 6.15)	0.879	0.379
TBIl^a^	7.20 (6.40, 16.10)	8.40 (6.45, 15.50)	−0.689	0.491
IBIL^a^	5.50 (3.60, 13.50)	6.20 (4.20, 11.50)	−0.594	0.553
MPV^a^	11.60 (10.20, 12.50)	11.60 (10.20, 12.50)	−0.227	0.820
Cr^a^	66.00 (57.00, 80.00)	65.00 (59.00, 78.50)	0.498	0.619
AST^a^	17.80(15.00, 21.60)	17.00 (14.00, 22.00)	0.229	0.819
ALT^a^	21.00 (11.00, 30.19)	21.00 (11.00, 30.00)	0.059	0.953
TG^a^	0.66 (0.57, 0.99)	0.68 (0.56, 1.095)	−0.328	0.743
TC^a^	4.45 (4.01, 5.17)	4.47 (4.01, 5.155)	−0.027	0.979
FIB^a^	3.01 (2.54, 3.76)	2.95 (2.58, 3.445)	0.948	0.343
WBC^a^	8.05 (6.57, 9.9125)	8.06 (6.57, 9.90)	0.129	0.897
Alb^a^	43.00 (37.00, 45.00)	43.00 (40.00, 46.00)	−1.597	0.110
**Gran** a	**6.22 (4.34, 7.86)**	**3.97 (2.63, 4.56)**	**9.171**	**< 0.001** *
Lym^a^	1.59 (1.15, 2.07)	1.54 (1.32, 2.38)	−1.401	0.161
**Plt** a	**251.47 (203.53, 277.97)**	**213.61 (179.41, 264.02)**	**3.472**	**0.001** *
**SII** a	**862.13 (676.05, 1061.35)**	**366.76 (256.72, 526.27)**	**11.895**	**< 0.001** *
**PNI** c	**50.61** ± **6.59**	**53.75** ± **4.81**	**4.72**	**< 0.001** *

*Note:* Systolic Blood Pressure at admission (SBP); Diastolic Blood Pressure at admission (DBP); Body Mass Index (BMI); Glycosylated Hemoglobin (HbA1C); Creatinine (Cr); Albumin (Alb); Aspartate Aminotransferase (AST); Alanine Aminotransferase (ALT); Total Bilirubin (TBIL); Indirect Bilirubin (IBIL); Triglyceride (TG); High‐Density Lipoprotein (HDL); Low‐Density Lipoprotein (LDL); Total Cholesterol (TC); White Blood Cell (WBC); Neutrophil (Gran); Lymphocyte (Lym); Platelet (Plt); Mean Platelet Volume (MPV); Fibrinogen (FIB).

^a^ indicated that the index does not follow a normal distribution and is expressed as the median and interquartile range.

^b^ indicated that the variable is a categorical variable and is expressed in percentage form.

^c^ indicated that the index follows a normal distribution and is expressed as mean ± SD.

* indicated that the difference had statistical meaning (*p* < 0.05).

### 3.2 Nutritional Immunity and the Occurrence of SSNHL

**Figure 1:**
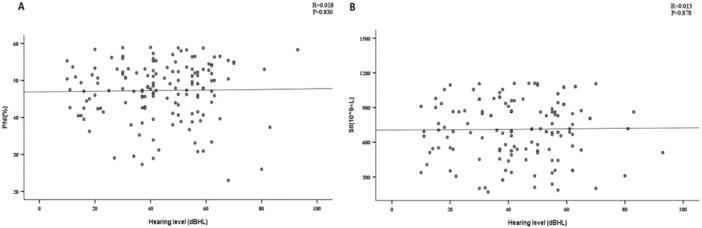
Scatter plot of correlation analysis for (A) PNI or (B) SNI and theseverity of SSNHL.

By plotting correlation scatter diagrams, we investigated the link between PNI, SII, and hearing loss. Figure 1 results indicated no correlation between hearing loss (as a continuous variable) and either PNI or SII (*R* = 0.018, *p* = 0.830; R = 0.013, *p* = 0.878), suggesting nutritional immunity levels are unrelated to SSNHL severity.

Subsequently, we integrated variables such as age, sex, BMI, blood pressure, and relevant laboratory parameters into the binary logistic regression model. The variance inflation factor test was applied to eliminate the collinearity of variables. The results in Table 2 showed that neutrophils (OR = 1.758, 95% CI: 1.402~2.205), PNI (OR = 0.878, 95% CI: 0.824~0.935), and SII (OR = 1.005, 95% CI: 1.004~1.007) were correlated with the occurrence of SSNHL (*p* < 0.001).

**Table 2:** Associations between the level of nutritional immunity and the occurrence of SSNHL were assessed using logistic regression analyses.

Variable	Univariate analysis			Multivariate analysis		
	OR	95% CI	*p* value	OR	95% CI	*p* value
Age	0.993	(0.964, 1.022)	0.623			
BMI	1.284	(1.01, 1.632)	0.041			
Sex	1.617	(0.745, 3.510)	0.225			
DBP	0.982	(0.912, 1.043)	0.975			
SBP	0.975	(0.947, 1.019)	0.982			
HDL	1.050	(0.291, 2.802)	0.860			
LDL	1.429	(0.836, 2.356)	0.200			
HbA1c	1.424	(0.864, 2.348)	0.166			
TBIl	0.953	(0.868, 1.048)	0.322			
IBIL	1.043	(0.941, 1.157)	0.419			
MPV	0.974	(0.748, 1.268)	0.843			
Cr	0.994	(0.971, 1.017)	0.599			
AST	0.987	(0.917, 1.062)	0.718			
ALT	0.978	(0.908, 1.053)	0.556			
TG	1.690	(0.679, 4.207)	0.260			
TC	0.768	(0.494, 1.193)	0.239			
FIB	0.932	(0.520, 1.669)	0.812			
WBC	0.994	(0.831, 1.189)	0.95			
Alb	0.938	(0.812, 1.083)	0.381			
Gran	1.731	(1.307, 2.294)	< 0.001	**1.758**	**(1.402, 2.205)**	**< 0.001** *
Lym	1.141	(0.461, 2.823)	0.776			
Plt	1.001	(0.992, 1.010)	0.84			
**SII**	1.006	(1.004, 1.009)	< 0.001	**1.005**	**(1.004, 1.007)**	**< 0.001** *
**PNI**	0.910	(0.872, 0.949)	< 0.001	**0.878**	**(0.824, 0.935)**	**< 0.001** *

* Indicated that the difference had statistical meaning (*p* < 0.05).

### 3.3 Nutritional Immunity and SSNHL Treatment Outcomes

**Figure 2:**
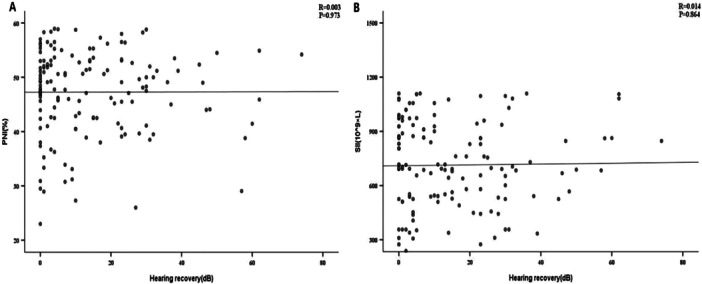
Scatter plot of correlation analysis for (A) PNI or (B) SNI and hearing recovery.

Patients with SSNHL were divided into an effective group (*n* = 62) and an ineffective group (*n* = 88) according to their levels of hearing recovery. Baseline and clinical characteristics were similar between the two groups, with no significant differences (*p* > 0.05). Interestingly, there were significant differences in SII and PNI (*p* < 0.001). The PNI levels were notably greater in the effective group than in the ineffective group (862.13 vs. 366.76), while SII levels were higher in the ineffective group compared to the effective group (53.75 ± 4.81 vs. 48.97 ± 7.20), as shown in Table 3. We then plotted a scatter plot of the correlation between SII, PNI and hearing recovery. The data in Figure 2 demonstrated that there was no correlation between PNI and SSNHL or between SII and SSNHL when hearing recovery was viewed as a continuous variable (*p* > 0.05). We then incorporated age, sex, BMI, blood pressure, and laboratory parameters into the binary logistic regression model. The variance inflation factor test was applied to eliminate the collinearity of variables. PNI and SNI are correlated with the prognosis of SSNHL. PNI (OR = 1.075, 95% CI: 1.013–1.137) and SII (OR = 1.004, 95% CI: 1.002–1.005) were risk factors for SSNHL (*p* < 0.01). The detailed results are shown in Table 4.

**Table 3:** Basic information of the effective and ineffective groups for SSNHL treatment.

	Effective group (*n* = 62)	Ineffective group (*n* = 88)	*t*/*Z*/*χ* ^*2*^	*p* value
**Baseline characteristics**				
Age	52.00 (38.00, 62.75)	49.00 (35.00, 60.50)	−0.752	0.452
BMI	22.41 (21.61, 24.92)	23.58 (22.36, 24.79)	1.519	0.129
Gender				
Male	31.00 (45.59%)	37.00 (54.41%)	0.929	0.335
Female	31.00 (37.80%)	51.00 (62.20%)		
DBP	74.00 (69.00, 82.25)	78.00 (74.00, 81.94)	1.550	0.121
SBP	118.00 (109.75, 128.00)	122.00 (115.00, 128.00)	1.384	0.166
**Clinical characteristics**				
Affected side	2.00 (1.00, 2.00)	1.50 (1.00, 2.00)	−0.194	0.846
Tinnitus	1.00 (1.00, 1.00)	1.00 (1.00, 1.00)	1.199	0.230
Vertigo	2.00 (1.00, 2.00)	2.00 (2.00, 2.00)	1.340	0.180
Ear fullness	2.00 (1.00, 2.00)	2.00 (1.00, 2.00)	0.448	0.654
Time to treatment	7.00 (6.00, 8.00)	7.00 (6.00, 8.00)	−0.222	0.825
PTA	50.50 (40.50, 58.75)	55.00 (38.00, 71.00)	1.190	0.234
**Laboratory indicators**				
HDL	1.35 (1.11, 1.72)	1.30 (1.08, 1.48)	−0.879	0.379
LDL	2.70 (2.38, 3.51)	3.15 (2.51, 3.44)	0.644	0.520
HbA1c	5.70 (5.40, 6.20)	5.70 (5.40, 6.70)	0.879	0.379
TBIl	8.30 (6.40, 16.10)	7.00 (6.25, 16.30)	−0.690	0.490
IBIL	6.10 (4.30, 12.38)	4.85 (3.35, 13.60)	−1.186	0.236
MPV	11.40 (10.20, 12.50)	11.70 (10.20, 12.50)	0.460	0.645
Cr	66.00 (57.00, 85.00)	65.00 (59.00, 79.50)	0.128	0.898
AST	17.30 (15.30, 21.60)	18.00 (15.00, 21.60)	−0.013	0.989
ALT	18.00 (10.00, 30.25)	22.00 (11.25, 30.56)	0.586	0.558
TG	0.64 (0.54, 0.98)	0.67 (0.59, 0.99)	0.808	0.419
TC	4.49 ± 0.97	4.51 ± 0.96	−0.123	0.900
FIB	2.95 (2.41, 3.48)	3.12 (2.58, 3.78)	1.694	0.090
WBC	8.08 (7.37, 9.20)	7.83 (6.19, 10.44)	−0.618	0.536
Alb	42.00 (34.75, 44.00)	42.00 (37.00, 45.00)	0.170	0.865
Gran	5.64 (3.02, 7.92)	6.31 (4.77, 6.94)	1.603	0.109
Lym	1.67 (1.13, 2.58)	1.59 (1.20, 2.00)	−1.149	0.250
Plt	243.19 (219.65, 294.11)	256.90 (183.29, 277.86)	−0.996	0.319
**SII**	**862.13 (676.05, 1061.35)**	**366.76 (256.72, 526.27)**	**5.931**	**< 0.001**
**PNI**	**48.97** ± **7.20**	**51.76** ± **5.90**	**−2.60**	**0.010**

**Table 4:** Associations between the level of nutritional immunity and the prognosis of SSNHL were assessed using logistic regression analyses.

Variable	Univariate analysis			Multivariate analysis		
	OR	95% CI	*p* value	OR	95% CI	*p value*
Age	0.993	(0.973, 1.013)	0.466			
BMI	1.084	(0.901, 1.306)	0.392			
Sex	1.378	(0.717, 2.649)	0.336			
DBP	1.034	(0.982, 1.089)	0.205			
SBP	1.023	(0.989, 1.059)	0.188			
Affected side	0.938	(0.489, 1.796)	0.846			
Tinnitus	1.736	(0.702, 4.292)	0.233			
Vertigo	1.634	(0.796, 3.354)	0.181			
Ear fullness	1.178	(0.576, 2.409)	0.576			
Time to treatment	0.971	(0.812, 1.162)	0.752			
HDL	0.572	(0.208, 1.572)	0.279			
LDL	1.076	(0.752, 1.539)	0.689			
HbA1c	1.023	(0.686, 1.526)	0.911			
TBIl	0.995	(0.945, 1.047)	0.846			
IBIL	0.993	(0.932, 1.057)	0.815			
MPV	1.049	(0.841, 1.308)	0.607			
Cr	0.999	(0.978, 1.020)	0.917			
AST	0.986	(0.921, 1.056)	0.691			
ALT	1.007	(0.978, 1.037)	0.647			
TG	1.071	(0.517, 2.219)	0.854			
TC	1.022	(0.728, 1.436)	0.899			
FIB	1.438	(0.905, 2.284)	0.124			
WBC	1.024	(0.893, 1.176)	0.713			
Alb	1.073	(1.008, 1.141)	0.027			
Gran	1.201	(1.028, 1.402)	0.021			
Lym	1.013	(0.970, 1.057)	0.565			
Plt	0.996	(0.989, 1.002)	0.202			
**SII**	**1.004**	**(1.002, 1.005)**	**< 0.001**	**1.004**	**(1.002, 1.005)**	< 0.001*
**PNI**	**1.071**	**(1.015, 1.129)**	**0.012**	**1.073**	**(1.013, 1.137)**	0.017*

**Figure 3:**
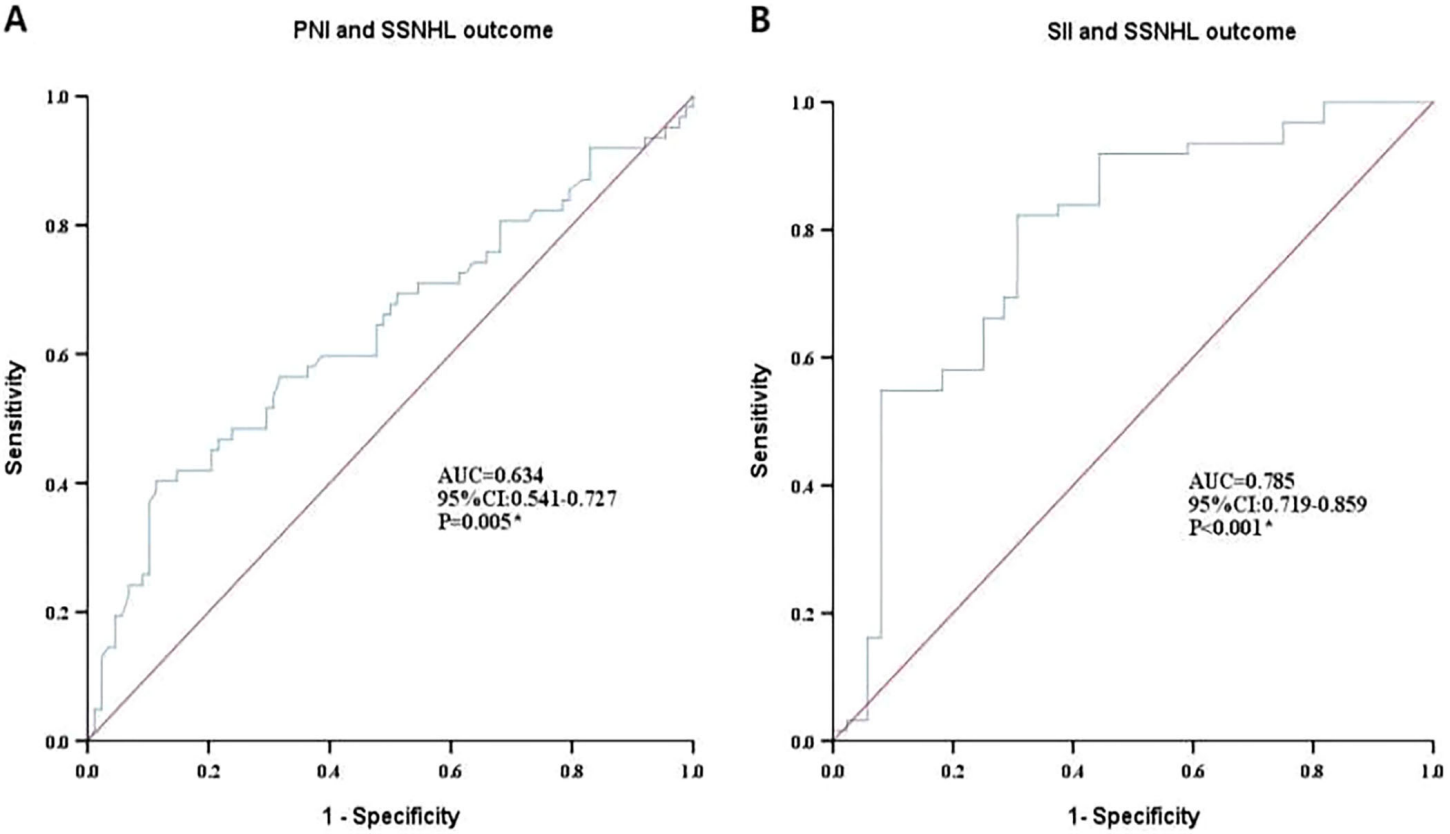
ROC curve of the prognostic model for (A) PNI or (B) SII.

ROC curve analysis in Figure 3 was used to examine the prognostic model performance. The model evaluation results showed that the AUC of the prognostic model for PNI or SII and SSNHL were 0.634 (95% CI: 0.541–0.727) and 0.785 (95% CI: 0.719–0859), respectively, indicating that the prognostic model had good predictive value (*p* < 0.01), suggesting that the level of PNI or SII can be used as predictor of SSNHL prognosis.

## 4 Discussion

Hearing loss and recovery could be greatly influenced by the body's inflammation and nutritional status [11]. The recovery rate (41.3%, 62/150) aligns with findings from multicenter studies in China. For instance, Zhang et al. [22] reported a 42.6% efficacy rate in unilateral SSNHL patients, suggesting regional and cohort‐specific variations. Differences may stem from our strict inclusion criteria (e.g., excluding patients with prior treatments or comorbidities), which likely contributed to a more homogeneous but potentially lower‐recovery subgroup. Despite being a single‐center study, our cohort's baseline demographics (age, sex distribution) are consistent with Chinese epidemiological data [23]. Additionally, SII and PNI thresholds in our study match prior reports [24, 25], supporting the validity of core conclusions. This study encompassed all subtypes of patients with SSNHL. Our findings revealed that the SSNHL group exhibited markedly elevated SII and neutrophil levels, alongside a reduction in PNI, compared to the control group. Moreover, SSNHL patients deemed effective had notably higher PNI levels compared to the ineffective group. These experimental results indicate that SII and PNI may serve as effective predictors for the occurrence and prognosis of SSNHL.

SSNHL represents a prevalent emergency within the field of otorhinolaryngology. It is characterized by an abrupt onset of hearing impairment, dizziness, and tinnitus, occurring within minutes to hours, and its etiology remains largely unidentified. This condition poses significant threats to patients' occupational and daily activities [8]. The incidence of SSNHL is increasing, potentially linked to the evolving lifestyles of contemporary society. Although the precise etiology of SSNHL remains undetermined, current evidence suggests that immunoinflammatory responses and microvascular disturbances are the most plausible pathophysiological mechanisms. The cochlea, which is supplied by the labyrinthine artery—a terminal artery devoid of collateral circulation—exhibits a high metabolic demand, rendering it particularly susceptible to ischemic and hypoxic insults [26]. Bacterial or viral infections can induce cochlear endothelial dysfunction or vasospasm leading to SSNHL, and a number of cardiovascular risk factors can also induce cochlear microvascular embolism, thrombosis, or increased blood viscosity leading to SSNHL [27, 28]. Results of several multicenter clinical studies have shown that SSNHL may be associated with inner ear circulation disorders [29, 30].

Malnutrition and immune dysfunction are believed to be closely related to the occurrence and recovery of hearing loss [14]. Specifically, elevated systemic SII reflects heightened pro‐inflammatory cytokine activity (e.g., IL‐1β, TNF‐α) that exacerbates cochlear vascular endothelial damage [31], while NLRP3 inflammasome activation in the cochlea amplifies inflammatory cascades, leading to hair cell degeneration [32]. Concurrently, poor nutritional status, such as hypoalbuminemia, impairs neural repair and antioxidant defenses [33], whereas adequate nutrition may mitigate inflammation‐driven oxidative stress through regenerative pathways [34]. These mechanistic insights align with our clinical observations of elevated SII and reduced PNI in SSNHL patients, further supporting their utility as prognostic biomarkers. Neutrophils are often considered as inflammatory markers of cardiovascular disease. Previous studies have reported that elevated neutrophil counts are an effective indicator of poor prognosis in SSNHL [35]. Platelets play an important role in atherosclerosis and may cause microcirculatory disorders [36]. SII serves as a marker indicating the immune and inflammatory condition of the body, derived from lymphocyte, neutrophil, and platelet counts. Earlier research has indicated that SII is crucial in forecasting the outcomes of tumors and inflammatory conditions [37]. Furthermore, a recent retrospective study discovered a strong link between the SII value and poor prognosis in SSNHL [38]. PNI is an indicator that reflects the nutritional level and is calculated by albumin and lymphocyte counts. Therefore, detecting SII and PNI levels will be helpful in evaluating the status and prognosis of SSNHL patients.

Our experimental findings showed that neutrophil and platelet counts were notably higher in the SSNHL patient group than in the control group, aligning with previous research [39]. Meanwhile, the SII value was statistically higher than that of the control group, and the PNI was significantly lower. This result suggests that the inflammation level in SSNHL patients is increased and the nutritional level is decreased. Previous study has reported that inflammatory responses can affect hearing by reducing the nutritional levels [40]. This is consistent with our finding that lower PNI values and higher SII values may be potential markers for the development of SSNHL.

In addition, recent findings suggest that orally administered micronutrients can improve hearing in cochlear inflammation treatment [41]. Our experiments revealed that the effective group had notably higher PNI levels and significantly lower SII values than the ineffective group. This result suggests that higher PNI values and lower SII values may be associated with a better prognosis for SSNHL.

After calculating an ROC curve, the model was evaluated for its predictive capabilities. We believe that SII and PNI can be used as effective indicators for predicting the prognosis of SSNHL. Based on the above experimental results, we suggest that SSNHL patients need to receive anti‐inflammatory and nutritional therapy to improve the prognosis. However, conventional corticosteroids have certain side effects. For patients with high SII value and low PNI value, alternative treatment options can be considered, such as intravenous hormone injection (3 days), improved nutritional intake and increased physical exercise [42].

PNI and SII can be quickly calculated through routine blood tests. The calculation of SII and PNI values allows clinicians to more thoroughly assess the inflammation and nutritional status in patients with SSNHL. In addition, these indicators are crucial for early diagnosis, medical care and improved prognosis of SSNHL patients. Clinicians can develop personalized treatment plans while evaluating the condition of SSNHL patients.

To the best of our knowledge, no previous studies have examined the potential beneficial effects of nutritional levels on patients with SSNHL. Our research aims to provide novel insights into the future management of SSNHL. However, this study has some limitations. Firstly, it is conducted at a single center with a relatively small number of participants, which might lead to selection bias even though strict inclusion and exclusion criteria were applied. Secondly, hematological indicators were collected only at the time of admission, resulting in a lack of continuous observational data. Additionally, the correlation between PNI values or SII values and hearing outcomes was relatively weak, suggesting that other factors may contribute to the pathogenesis of SSNHL. Further research is required to explore the relationship between inflammatory status, nutritional levels, and SSNHL more comprehensively.

This study underscores the significant implications of the PNI and SII in patients with SSNHL. The findings indicate that these indices have the potential to serve as valuable tools in clinical practice. Specifically, PNI and SII may be employed as early screening instruments to identify high‐risk patients, facilitating timely interventions that could improve treatment outcomes. Moreover, by evaluating PNI and SII levels, clinicians can devise personalized treatment plans that are tailored to the nutritional and immune status of individual patients, thereby optimizing therapeutic strategies. Regular monitoring of these indices during treatment could also yield critical insights into therapeutic efficacy, allowing for necessary adjustments in treatment protocols. Additionally, given the correlation between PNI, SII, and patient prognosis, these indices could be integrated into routine clinical assessments to provide more precise prognostic information. It is also recommended to monitor the dynamic changes of PNI/SII as well as the changes in hearing recovery over time (e.g., 3 to 6 months after treatment). Overall, the incorporation of PNI and SII in clinical settings holds promise for enhancing the management and outcomes of SSNHL patients, meriting further investigation in future research endeavors.

## 5 Conclusion

In this study, we examined the connection between human inflammatory status, nutritional levels, and the occurrence and prognosis of SSNHL patients. Lower PNI values and higher SII values may promote the occurrence of SSNHL, while higher PNI values and lower SII values may suggest a better prognosis for SSNHL. Our findings will benefit clinicians in the disease management of SSNHL patients and in the development of individualized treatment plans.

## Author Contributions

**Xu Zhang:** conceptualization, data curation, formal analysis, investigation, methodology, project administration, validation, visualization, writing – original draft. **Wentao Zhang:** data curation, formal analysis. **Maohua Wang:** investigation, methodology. **Bing Guan:** supervision. **Qiao Jiang:** formal analysis, investigation, supervision, writing – review and editing. **Chunping Yang:** investigation, resources, software, supervision, validation, visualization, writing – review and editing.

## Ethics Statement

The study was approved by the Ethics Committee of the Ethics Review Committee of the Northern Jiangsu People's Hospital Affiliated to Yangzhou University (Approval No. 2023ky051) and conducted according to the Declaration of Helsinki.

## Conflicts of Interest

The authors declare no conflicts of interest.

## Supporting information

Supporting Figure S1.

## Data Availability

The datasets generated and analyzed during the current investigation are available upon reasonable request from the corresponding author.
