# Supplementary figures and images for: Utility of Prognostic Nutritional Index and Systemic Immune‐Inflammation Index Inpatients With Sudden Sensorineural Hearing Loss: A Large Prospective Cohort Study
Source: Immun Inflamm Dis. 2025 Jun 17;13(6):e70217. doi: 10.1002/iid3.70217 (PMC12171996; doi:10.1002/iid3.70217)

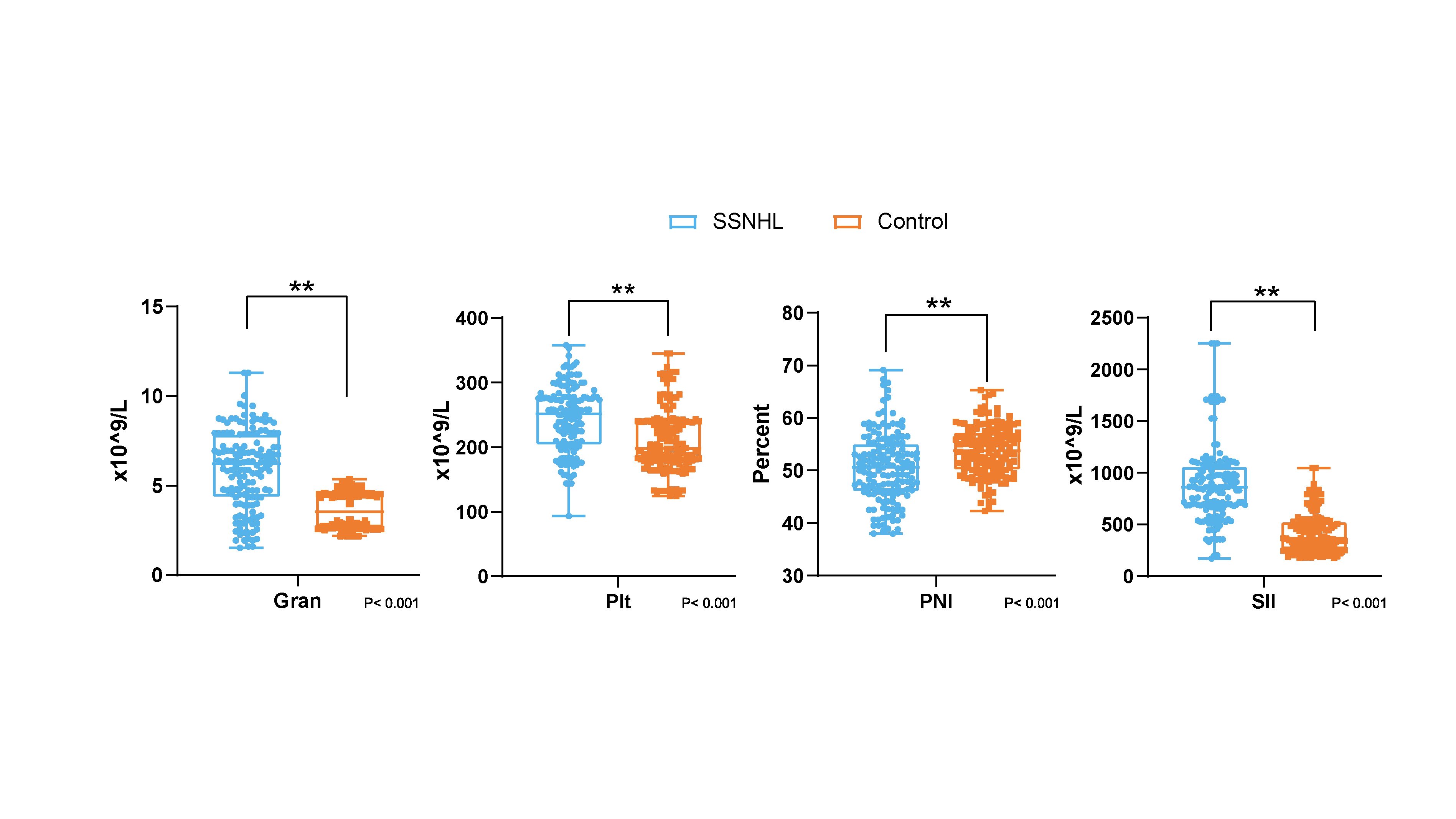

Supplement: Supplementary file 1 — Supporting Figure S1. [file IID3-13-e70217-s001.tiff]
